# Fur on the Tongue

**Published:** 1880-11

**Authors:** Dyce Duckworth

**Affiliations:** St. Bartholomew’s Hospital Reports


### FUR ON THE TONGUE.

Mr. Henry T. Butlin, (St. Bartholomew's Hospital Reports) says that his observations have convinced him that it is composed, in health and disease, chiefly of minute living organisms "Schistomycetes," and not, as is generally supposed, of cast-off epithelium, although this is always, and the debris of food, generally, detected in it.

The organisms of which the fur consists may, he says, enter the mouth with the inspired air or with solid or liquid food. Once arrived in the mouth, it is difficult to imagine a better nidus than the surface of the tongue affords for their support and development. During the day the tongue in health is continually cleansed by being rubbed against the roof of the mouth and against the gums and teeth. During the night, on the contrary, the cleansing of the tongue is not nearly so efficiently carried on; early in the morning, therefore, the coating of fur is thicker than at any other time of day. The fur is always thickest at the center and back part of the tongue, which are precisely the parts which are least movable and hence most difficult to cleanse.

He regards cases of unilateral furring as peculiarly illustrative of the laws of furring. In two cases which come under his observation, the left half of the tongue was coated three or four times as thickly as the right. the difference being due, in each case, to the fact that the left upper jaw had been removed only a few weeks previously. so that most of the cleansing operations was absent from that side.

Mr. Butlin explains the occurrence of furring of the tongue after a heavy meal or an excess in wine, as follows: Under these circumstances, he says, the breathing is oppressed, and the mouth is generally kept open. This favors the access of the parosite to the tongue, which becomes dryer than usual, but not generally so dry that the development of the fungus is interfered with. The movements of the tongue are consequently less active—a condition which favors the accumulation of fur.

Unfortunately, the examination of the fur on the tongue will afford us no aid in diagnosis, since the author has not been able to discover that any one of the organisms he has described, as occurring in fur, is constantly met with in any disease. Certain forms, it is true, are more commonly seen in certain diseases than in others, but not with sufficient frequency to justify us in relying upon their presence or absence in coming to a conclusion, as to the nature of the disease we have to deal with.

### GINGIVITIS IN CASES OF CONGENITAL DISEASE OF THE HEART.

In one of his communications the author calls attention to a peculiar ulcerative form of gingivitis met with in cases of congenital disease of the heart. In the cases which have come under his observation the ulceration has commonly occupied the lower gums, about the incisor teeth.

In one case, which he describes in detail, "there was loss of substance, and the teeth were a good deal exposed. The surface was very red, raw and fungus looking; it bled readily. Shreddy, sloughing particles were more or less adherent to it. It was painful during mastication. There was no general sponginess of the gums, and no other form of stomatitis present."

The case proved rebellious to several methods of treatment, but at length gradually improved under the use of borax and chlorate of potash. The author regards this ulceration as so characteristic that he has been led by its presence to seek for evidences of cardiac defect.—*Dr. Dyce Duckworth, in St. Bartholomew's Hospital Reports.*

